# To Dr. Bradley

**Published:** 1804-01-01

**Authors:** R. Winterbottom

**Affiliations:** South Shields


### To Dr. BRADLEY.

Sir,

I Should not have again troubled you upon a subject, which has probably ere this exhausted your patience; but the fear lest my silence should be attributed to the consciousness of having acted with duplicity, induces me to offer a justification of my conduct. In a paper upon Apoplexy, plexy, inserted in No. 51, of the Med. Journal, Dr. Mossman, reverting to the dispute respecting the employment of emetics in that disease, observes (Dr. Cullen) "in his treatment of the disease, if he do not positively forbid the exhibition of emetics, (about the employment of which there has been so much Controversy) he very certainly does not recommend them. *A quotation, indeed, from the MS. lectures of Dr. Cullen, admitting of the use of emetics, has been communicated to us anonymously; but as it comes in this shape, it ought not to have been given at all, for it is not evidence.*"

Dr. M. appears to me to be extremely fastidious in making this objection, though I do not feel offended at the insinuation it conveys, because, to use a *homely proverb*, "Nothing hurts but the truth." But as it is incumbent upon me to remove every suspicion of deceit, I pledge myself to give Dr. M. any satisfaction he chooses respecting the accuracy of the quotation alluded to, which is as follows. "This (vomiting, Dr. Cullen observed) hath sometimes been practised with safety. It is difficult to explain how full vomiting is employed without increasing the congestion; yet here, facts are in part against our theoretic fears." From this quotation, I think, the Reader can only infer, that Dr. Cullen would not have *censured* the exhibition of an emetic under proper circumstances. If, however, the quotation be false, and if I have recorded words which Dr. Cullen never uttered, I may easily be contradicted by numbers of gentlemen who attended the Lectures of *our* "justly celebrated Preceptor," in the years 1787—88. With regard to my having written anonymously, it surely can be of no great consequence to the Reader, in the present instance, where equal obscurity would prevail, whether the real signature or a fictitious one be subscribed.

Notwithstanding what has been said upon the use of emetics in Apoplexy, and the respectable authorities which have been adduced in support of their safety, at least, Dr. M. adheres strenuously to his former opinion, that the practice is fraught with danger. It is remarkable also, that Dr. M. has not alleged any plausible reason for his deep-rooted antipathy; and that he has not informed us what share the emetics, exhibited in the two instances which he reports, were supposed to have had in accelerating the death of the patients. An emetic, Dr. M. observes, is an *awful instrument*; and so, it may be added, is an enema,

 yet yet it is frequently administered by the old ladies with great success.

Though I am ready to acknowledge the very beneficial effects derived from the prudent use of the lancet in Apoplexy, I cannot agree with Dr. M. in thinking that 'every man who has treated apoplexy must have witnessed *instant relief* by the operation of bleeding, (Medical Journal, vii. 208); neither can I admit with him, that 'the morbid-phenomena of this disease, both before and after death, are so uniform, as to draw from *every author* a description very exactly similar.' Very diffeient indeed are the histories of dissections in this disease; but I forbear entering into particulars, as I do not wish to have the Controversy renewed. *Requiescat in pace.*

Before I quit the subject, however, I cannot refrain from expressing my disapprobation of the slighting manner in which Dr. M. speaks of Riverius and Van Helmont.— "There is (he says) a stateliness, I think, in quoting such authorities as Riverius or Van Helmont; and there is a want of fairness in deducing from the opinions of those men, any sound practical inferences." Does Dr. M. mean, that we should not consult medical records; and that the valuable observations which our Predecessors have bequeathed to us, should be neglected, as useless? Surely, some respect is due to age, some deference to those who have removed the thorns which obstruct our progress. The inordinate praise which some have lavished upon the antients, has been justly ridiculed; but pedantry appears to me to be more excusable than that democratic spirit which endeavours to strip them of their justly-acquired honours. Indeed, when I perceive this spirit of detraction prevailing to a great degree, I am tempted to a certain little fable of Esop's, respecting the fox in the vineyard. I do not wish to offend Dr. M.; neither do I presume to inform him of the characters of the men whom he treats so disrespectfully. Riverius, it is well known, was no disgrace to a Professor's chair in an University which has ever boasted of her learned men. He was an excellent observer; and the remedy which still retains his name, and which we are daily obliged to have recourse to, is no despicable memorial of his practical skill. The treatment which the memory of Van Helmont has received, ought to caution physicians from indulging in idle speculations. It has been too much the custom for the witlings of succeeding ages, to use this venerable name as a *butt* to shoot their puny shafts at; but men of discernment have done justice to to his genius, his learning, and his industry. "The friend of truth, says a learned writer, (Sprengel Geschichte der Arzneykunde) contemplates with pleasure the writings of a man, who, though addicted to the superstitions of his age, detected innumerable theoretical and practical errors; and published observations, which later physicians, from a want of better information, have considered as recent discoveries. Helmont may be placed by the ignorant in the same rank with Paracelsus, and be despised; but impartial History will assign the wreath of merit to this neglected physician." The great and good Baron Haller bestows on Van Helmont a similar eulogium; he calls him "vir acuti ingenii, in detegendis aliorum erroribus acris, in colligendis eventis suæ causæ faventibus ingeniosus, non expers anatomes, &c." Biblioth. Med. Pract.

R. WINTERBOTTOM, M. D.

*South Shields, Aug.* 20, 1803.

